# Rapid and Sensitive Detection of *Salmonella* via Immunomagnetic Separation and Nanoparticle-Enhanced SPR

**DOI:** 10.3390/microorganisms13081914

**Published:** 2025-08-16

**Authors:** Fengzhu Liang, Yuzhen Li, Yan Cui, Jianhua Zhang

**Affiliations:** 1 School of Agriculture and Biology, Bor S. Luh Food Safety Research Center, Shanghai Jiao Tong University, Shanghai 200240, China; fzliang@sjtu.edu.cn (F.L.); cyan9028@163.com (Y.C.); 2 College of Food Science and Technology, Jilin Agricultural University, Changchun 130118, China; 3 NMPA Key Laboratory for Testing Technology of Pharmaceutical, Shanghai Institute for Food and Drug Control, Shanghai 201203, China

**Keywords:** *Salmonella* Typhimurium, capture efficiency, surface plasmon resonance, sensitivity, gold nanoparticles

## Abstract

The widespread prevalence of *Salmonella* underscores the urgent need for rapid, sensitive, and reliable detection methods to ensure food safety and protection of public health. In this study, we successfully developed an integrated detection system that combines immunomagnetic separation with surface plasmon resonance (SPR) analysis. This system achieved high capture efficiencies, exceeding 96.04% in phosphate-buffered saline and over 91.66% in milk samples artificially spiked with *S.* Typhimurium at concentrations below 4.2 × 10^4^ CFU/mL. However, direct SPR detection of the isolated *S*. Typhimurium showed limited sensitivity, with a limit of detection (LOD) of 4.2 × 10^7^ CFU/mL. Incorporating a sandwich assay with antibody-conjugated gold nanoparticles significantly enhanced sensitivity, lowering the LOD by six orders of magnitude to 4.2 × 10^1^ CFU/mL. The whole integrated process, integrating immunomagnetic separation with SPR analysis, was completed within 50 min. These results demonstrate that this AuNP-enhanced SPR platform offers both the rapidity and sensitivity essential for effective monitoring of food safety and traceability in *Salmonella*-related foodborne outbreaks, particularly in products such as milk.

## 1. Introduction

*Salmonella* is a major global foodborne pathogen, causing over 93 million infections annually [1]. *S*. Typhimurium is among the most prevalent serotypes worldwide [2,3,4] and has consistently ranked in the top two in China over the past several years [5,6]. However, its typically low abundance and uneven distribution in food samples pose substantial challenges for detection. A key obstacle in food safety monitoring is the reliable identification of trace *Salmonella* contamination within complex food matrices.

This underscores the need for highly sensitive detection methods capable of (1) effectively separating and concentrating *Salmonella* from complex matrices such as dairy, meat, and eggs and (2) accurately identifying the pathogen [7]. Reliable detection platforms are particularly critical for ready-to-eat foods, where rapid traceability of *Salmonella* contamination is essential for outbreak prevention and public health protection [8,9,10].

Regardless of the detection method, effective separation and concentration of target bacteria from food matrices remain essential. Conventional microbiological protocols typically involve 24–48 h incubation in selective media for enrichment [11]. In contrast, rapid approaches such as centrifugation, filtration, and the use of magnetic nanoparticles [12,13,14,15,16] can reduce enrichment time. Among these techniques, immunomagnetic separation (IMS) has been widely adopted for pathogen isolation, concentration, and detection. IMS employs superparamagnetic nanobeads functionalized with specific biorecognition molecules to selectively capture microorganisms, enabling rapid and highly specific enrichment under a magnetic field. For example, IMS has isolated *Salmonella* from skim milk within 35 min [15] and achieved over 95% capture efficiency (CE) for *E. coli* O157:H7 in just 20 min, with the added advantage of bead regeneration and reuse [14]. Moreover, under low-contamination conditions, an automated IMS system demonstrated 15.5% higher sensitivity in detecting *Salmonella* in poultry meat samples compared to the conventional broth-based enrichment methods [17].

Common analytical methods for detecting *Salmonella* include plate counting, molecular biology, immunoassays, and biosensor-based approaches. However, each of these methods presents certain limitations. Plate counting and real-time PCR, while reliable, are often time-consuming and laborious [11]. Immunoassay methods, such as colloidal gold immunochromatography [18], fluorescence immunochromatography [19], and enzyme-linked immunosorbent assay (ELISA) [20], typically involve multi-step procedures, rely on enzymatic labeling, and are susceptible to antibody cross-reactivity, which can lead to false-positive results and reduced specificity.

In recent years, biosensors have emerged as advanced platforms for *Salmonella* detection, offering rapid response times, high sensitivity, and strong specificity compared to conventional techniques [21,22,23]. Among them, optical biosensors are the most widely utilized class and show significant potential for addressing the stringent requirements of specialized applications [24,25].

Within this category, surface plasmon resonance (SPR) stands out for its label-free and real-time monitoring of interactions between specific ligands and intact viruses or bacterial cells. Immunoassay-based SPR detection offers advantages, such as low reagent use, reduced costs, minimal environmental requirements, fast detection, high-throughput, compact integration, and automation. Incorporating microfluidic channels with precise fluid control, high surface area, and efficient antibody immobilization further enhances antigen–antibody binding, thereby reducing assay time and increasing sensitivity [26,27].

For instance, limits of detection (LODs) of 2.5 × 10^5^ cfu/mL for *S.* Typhimurium in spiked milk samples [28] and 1.4 × 10^4^ cfu/mL in PBS buffer [26] were separately achieved using polyclonal antibody-based SPR biosensors. Subsequently, direct SPR assays utilizing monoclonal antibodies for the detection of *S.* Typhimurium were developed, demonstrating LODs of 6.0 log CFU/mL in buffer [29] and 5.9 log CFU/g in romaine lettuce [30].

Modified SPR strategies have been introduced to improve analytical performance further. Notably, magnetic nanoparticle–antibody sandwich assays reduced the LODs to 4.7 log CFU/mL in buffer and 5.2 log CFU/g in romaine lettuce samples [31], as well as to 1.4 × 10^1^ CFU/mL in PBS buffer [26].

Localized surface plasmon resonance (LSPR) arises from the collective oscillation of conduction electrons on metal nanoparticle surfaces under specific light frequencies. Noble metal nanoparticles (NMNPs), particularly gold nanoparticles (AuNPs) and silver nanoparticles (AgNPs), play a pivotal role in LSPR to enhance signal sensitivity through the exploitation of LSPR effects [32,33,34,35]. AuNPs are especially favored for biosensing owing to their biocompatibility and ease of biomolecule conjugation [36].

For example, SPR with amine-functionalized AuNPs enabled immunoglobulin M detection at 0.08 ng/L [37], and Lao et al. achieved low LOD thrombin detection using 13 nm AuNPs [38]. LSPR has been applied to detect anti-*Brucella* antibodies [9], *E. coli* O157:H7 at 10 CFU/mL within two hours using AuNP–chicken antibody conjugates [39], and *S.* Typhimurium in pork at ~10^4^ CFU/mL within 30–35 min via an AuNP-based aptamer LSPR chip without pre-enrichment [32].

These results highlight the high sensitivity and versatility of AuNP-enhanced SPR. Building on prior work, this study combines antibody-functionalized immunomagnetic beads (Ab-IMBs) with AuNP-enhanced SPR to create a sensitive, specific platform for rapid *Salmonella* detection in complex samples like milk. Two sandwich formats (Ab-ST-Ab and Ab-ST-Ab-AuNPs) were optimized, both significantly boosting the SPR signal. The system enabled rapid and enrichment-free detection of *S.* Typhimurium with a lower LOD, outperforming conventional detection methods.

## 2. Materials and Methods

### 2.1. Strains and Cultural Conditions

*S*. Typhimurium ATCC14028, *Staphylococcus aureus* ATCC 29213, *Escherichia coli* ATCC 25922, *Bacillus subtilis* 168 CCTCC AB 130001, and *B. subtilis* natto CGMCC No.2801 were prepared in the Food Microbiology Laboratory, School of Agriculture and Biology, Shanghai Jiao Tong University, China. All strains were stored at −70 °C in Luria–Bertani (LB) broth supplemented with 30% (*v*/*v*) glycerol. Prior to use, all the strains were reactivated on LB agar plates by incubation at 37 °C for 24 h.

### 2.2. Binding and Characterization of IMBs

The magnetic bead–antibody (MB-Ab) conjugate was prepared by coupling carboxylated Fe_3_O_4_ magnetic beads (200 nm MBs, 10 mg/mL; PuriMag^TM^ G-COOH, PuriMag Biotech, Xiamen, China) with rabbit polyclonal anti-*Salmonella* antibody (4 mg/mL; ab35156, Abcam, Cambridge, UK). The conjugate was designed to specifically recognize *Salmonella* Enteritidis, *S*. Typhimurium, and *S*. Heidelberg, without binding to other *Enterobacteriaceae*. Conjugation followed the manufacturer’s protocol, using a bead-to-antibody mass ratio of 4:1. Freshly prepared EDC/NHS (0.4 M EDC and 0.1 M NHS, pH 6.0) served as the coupling agent. To block unreacted carboxyl groups, 0.1 M ethanolamine was added and incubated for 30 min. The resulting IMBs were resuspended in PBS containing 0.01% Tween-20 and stored at 4 °C. All mixing steps were conducted using a vortex stirrer (BE-100, Haimen, China).

The MB-Ab conjugate was confirmed by comparing the UV-Vis spectra of the beads before and after modification using a TU-1810 spectrophotometer (Beijing, China).

### 2.3. Separation of Bacterial Samples

*S.* Typhimurium was cultured in LB broth at 37 °C for 8–12 h. Bacterial cells were harvested by centrifugation at 1500× *g* for 5 min, washed thrice with PBS, and resuspended to a final optical density of OD_600_ = 0.3. Ten-fold serial dilutions were then prepared in PBS, with the lowest concentration reaching 4.2 × 10^1^ CFU/mL. The actual cell counts were determined using the plate count method. Capture efficiency (CE) was calculated by the following equation:

$$CE(\%)=\frac{\mathrm{Colony}\;\mathrm{count}\;\mathrm{in}\;\mathrm{magnetic}\;\mathrm{bead}\;\mathrm{resuspension}\;(\mathrm{CFU})}{\mathrm{Colony}\;\mathrm{count}\;\mathrm{in}\;\mathrm{magnetic}\;\mathrm{bead}\;\mathrm{resuspension}\;(\mathrm{CFU})+\mathrm{Colony}\;\mathrm{count}\;\mathrm{in}\;\mathrm{supernatant}\;(\mathrm{CFU})}\times 100\%$$

An aliquot of 50 μL of each concentration of *S*. Typhimurium suspension, either in PBS or artificially contaminated milk, was mixed with 12.5 μg of IMBs and vertically rotated for 30 min. The IMBs bound to *S*. Typhimurium were then separated using an external magnetic field generated by a magnetic stand. The number of captured bacteria on the IMBs, as well as the remaining bacteria in the supernatant, were quantified using the plate count method. *Staphylococcus aureus* ATCC 29213, *Escherichia coli* ATCC 25922, *Bacillus subtilis* 168 (CCTCC AB 130001), and *Bacillus subtilis* Natto (CGMCC No.2801) were used as negative controls.

To regenerate and reuse the IMBs, and to evaluate the dissociation efficiency of *S*. Typhimurium from the magnetic beads, bacteria-bound IMBs were subjected to vertically rotation for 5, 10, and 15 min in the following solutions: (1) 0.05 M glycine–HCl buffer, pH 3.0; (2) 0.1 M citric acid–NaOH–HCl, pH 3.0; (3) 0.1 M citric acid–sodium citrate, pH 3.0; (4) 0.2 M phosphoric acid–sodium citrate, pH 3.0; (5) 4 M MgCl_2_; (6) 0.2 M citric acid–HCl, pH 3.0; (7) 10 mM NaOH; and (8) 0.85% phosphoric acid. After elution, the pH of each solution was adjusted to 7–8 using Tris–HCl (pH 8.0).

The number of bacteria remaining on the IMBs and those released into the eluent were quantified by the plate count method to calculate desorption efficiency. To assess recovery efficiency, 50 μL *S*. Typhimurium at 4.2 × 10^3^ CFU/mL was rotatably mixed with the regenerated IMBs, which had been stored in PBS at 4 °C following elution with each tested solution.

### 2.4. Synthesis of AuNPs

AuNPs with an average diameter of approximately 30 nm were synthesized by the trisodium citrate reduction method [40]. The sizes and optical properties of the AuNPs were characterized using a nanoparticle size analyzer (Zetasizer Nano S, Malvern, UK) and a UV-Vis spectrophotometer (TU-1810, 200–750 nm), respectively.

### 2.5. Preparation of the AuNP-Ab Conjugate

The AuNP-Ab conjugate was prepared as follows: 6 μL of antibody (400 μg/mL) was added dropwise to 600 μL of AuNPs suspension (pH 7.5) with gentle vortexing for 10 min at room temperature. Subsequently, a BSA solution was added to a final concentration of 1% and incubated for 30 min to block non-specific binding sites. The mixture was centrifuged at 6000× *g* for 30 min, and the supernatant was discarded. The resulting conjugate was washed thrice with 1% BSA solution.

To confirm successful conjugation, UV–visible spectroscopy was performed to compare the spectra of the AuNPs before and after antibody binding.

### 2.6. Fabrication of the Immunosensor and SPR Detection

A gold chip (CM5, Cytiva, America) was mounted onto the SPR instrument (Biacore 8K, GE Healthcare, IL, USA). Freshly prepared EDC/NHS was injected to activate the carboxyl groups, followed by injection of antibody solution (20 μg/mL, diluted in sodium acetate buffer, pH 5.0) at 10 μL/min for 300 s to achieve covalent immobilization. After washing with PBS, 1 M ethanolamine was injected at 30 μL/min for 180 s to block residual active carboxyl groups, followed by a final PBS rinse.

A reference flow cell treated with PBS instead of antibody served as a baseline control. SPR responses at each step were recorded in real time using BiacoreTM 8K Control software at room temperature.

**Figure 1:**
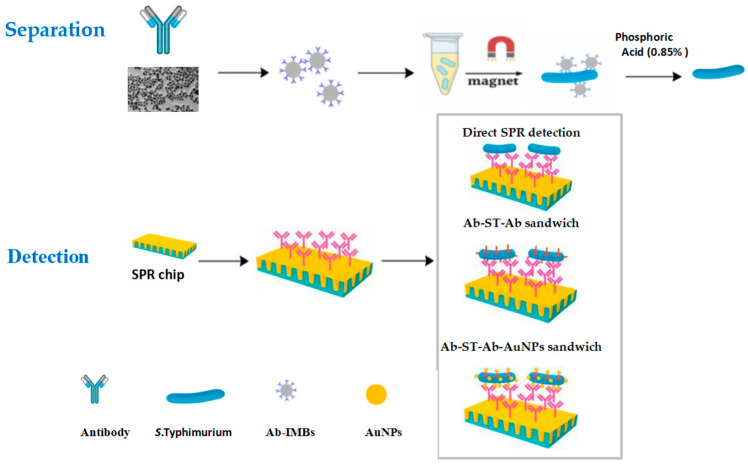
Schematic diagram of immunomagnetic bead (IMB)-based separation and AuNP-enhanced SPR sandwich detection of *Salmonella*.

Sandwich immunoassaies were employed for the detection of *S.* Typhimurium (Figure 1). The bacterial suspension was first injected onto the sensor chip, followed by a PBS wash to remove unbound components. Subsequently, either the antibody or the AuNP-Ab conjugate was injected for 5 min to complete the sandwich complex. To regenerate the sensor surface, 90 μL of 0.85% phosphoric acid was injected for 3 min.

All injections were performed at a flow rate of 10 μL/min, except for the blocking and regeneration steps, which were performed at 30 μL/min.

To evaluate the specificity of the *S*. Typhimurium biosensor, signal responses were measured for *Bacillus subtilis* 168, *B. subtilis* natto (CGMCC2801), *Staphylococcus aureus* (ATCC29213), and *Escherichia coli* (ATCC25922), each at 1.0 × 10^8^ CFU/mL. A *S*. Typhimurium sample at 4.2 × 10^1^ CFU/mL served as the positive control.

Sensitivity was assessed by testing *S*. Typhimurium samples at concentrations ranging from 4.2 × 10^1^ to 4.2 × 10^8^ CFU/mL.

### 2.7. Statistical Analysis

Quantitative enumeration data were log-transformed prior to analysis to stabilize variances. Linear regression was employed to analyze *Salmonella* enumeration results. Statistical analysis of the ultraviolet–visible absorption spectra and SPR response signals was conducted using Origin 9.1 (Origin Lab Corp., Northampton, MA, USA).

## 3. Results

### 3.1. Capture Efficiency of Bacterial Samples

**Figure 2:**
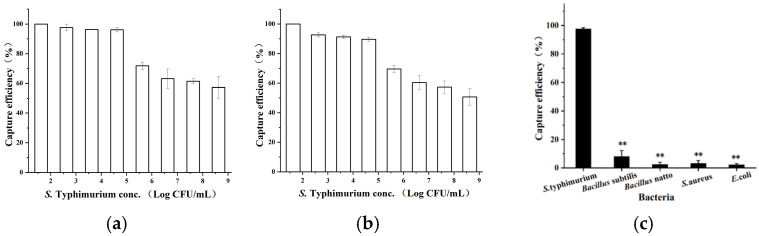
Capture efficiencies of MBs for *S*. Typhimurium across concentrations ranging from 4.2 × 10^1^ to 4.2 × 10^8^ CFU/mL in (**a**) PBS buffer and (**b**) artificially contaminated milk and (**c**) for *S*. Typhimurium (4.2 × 10^3^ CFU/mL) and four non-target bacteria (*B. subtilis* natto, *S. aureus*, *B. subtilis* 168, and *E. coli)* in artificially contaminated milk. ** *p* < 0.01.

As shown in Figure 2, the CE of *S.* Typhimurium in both PBS buffer and artificially contaminated milk consistently exceeded 96.04% and 91.66%, respectively, at bacterial concentrations below 4.2 × 10^4^ CFU/mL.

At a concentration of 4.2 × 10^3^ CFU/mL in spiked milk, 96.04 ± 1.49% of *S*. Typhimurium was specifically captured by the IMBs. In contrast, the CEs for *Bacillus subtilis* 168, *E. coli*, *B. subtilis* natto, and *Staphylococcus aureus* were significantly lower, standing at 7.99 ± 4.32%, 2.50 ± 1.53%, 3.24 ± 2.07%, and 2.20 ± 0.89%, respectively, demonstrating the high specificity of the system for *S*. Typhimurium.

**Figure 3:**
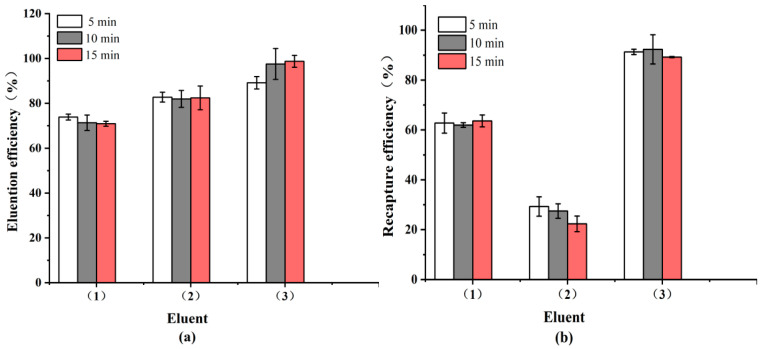
Effects of different eluents and elution times on (**a**) elution efficiency and (**b**) recapture efficiency of IMBs bound to *S.* Typhimurium: (1) 0.05 M glycine-HCl buffer, pH 3.0; (2) 0.1 M citric acid-NaOH-HCl, pH 3.0; (3) 0.85% phosphoric acid.

In addition to specificity, effective recycling requires that target bacteria be efficiently dissociated from the IMBs without compromising antibody integrity. In this study, eight eluents were evaluated for their dissociation performance. After 5 min of treatment, the top three eluents, 0.85% phosphoric acid, 0.1 M citric acid, and 0.05 M glycine-HCl buffer, achieved desorption efficiencies of 89.19 ± 2.76%, 82.76 ± 2.21%, and 73.87 ± 1.31%, respectively (Figure 3). The corresponding IMB recovery efficiencies were 92.32 ± 5.88%, 29.31 ± 3.89%, and 63.61 ± 2.39%. These results indicate that 0.85% phosphoric acid is the most effective eluent, providing both high CE and excellent IMB recovery, thereby preserving the functional integrity of the system.

### 3.2. Characterization of AuNP-Ab Conjugate

**Figure 4:**
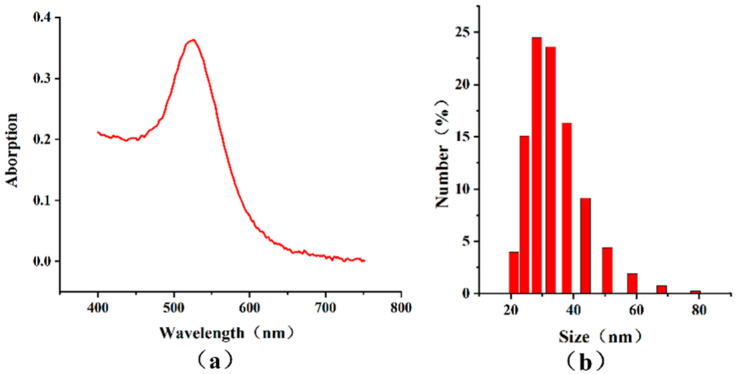
Characterization of AuNPs. (**a**) The visible light absorption spectrum and (**b**) particle size distribution.

The colloidal AuNPs displayed a characteristic bright red color, with an absorption peak at 527 nm in the visible light spectrum (Figure 4a). The average particle diameter was 33.47 ± 9.11 nm (Figure 4b).

**Figure 5:**
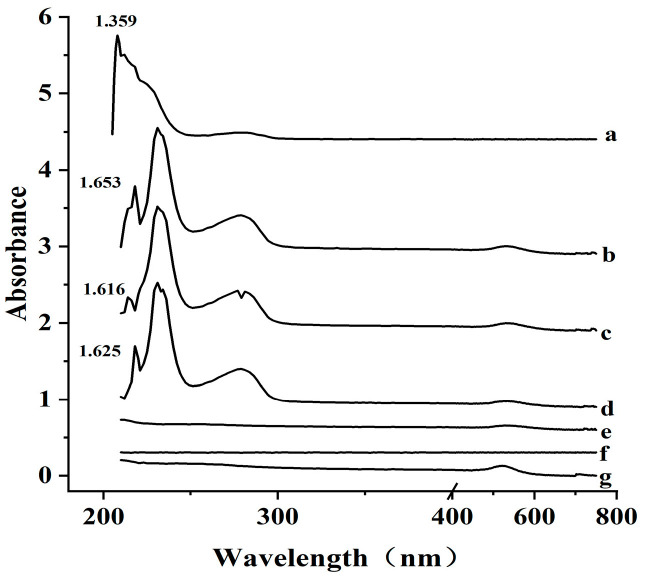
UV–Vis absorption spectra of (a) free Ab; (b–f) Ab-AuNP conjugate prepared with decreasing Ab concentrations (4.0, 3.2, 2.4, 1.6, and 0 μg, respectively); and (g) bare AuNP. Absorbance values at characteristic peaks are indicated.

The UV-Vis spectra of the antibody, AuNP, and AuNP-Ab conjugate in the range of 200–750 nm are shown in Figure 5. While AuNP exhibited no significant absorption in the 200–400 nm region, the antibody showed two distinct absorption peaks at 230 and 278 nm. When the amount of antibody used for labeling exceeded 2.4 μg, the resulting AuNP-Ab conjugate exhibited the same characteristic absorption peaks as the free antibody. This observation confirms the successful conjugation of antibody to AuNP, indicating the effectiveness of the labeling process.

### 3.3. Direct and Sandwich Strategies for SPR Detection of S. Typhimurium

To enable direct comparison, SPR curves at various concentrations were baseline-corrected using the negative control and plotted together. The LOD was defined as the lowest concentration consistently yielding a detectable resonance angle shift above baseline noise.

Direct SPR detection of *S.* Typhimurium exhibited a concentration-dependent response, with a moderate signal at 4.2 × 10^7^ CFU/mL and a pronounced response at 4.2 × 10^8^ CFU/mL. No signal was detected below 4.2 × 10^6^ CFU/mL, indicating effective sensor regeneration. However, the current LOD of 4.2 × 10^7^ CFU/mL may not meet the sensitivity demands of the food industry.

**Figure 6:**
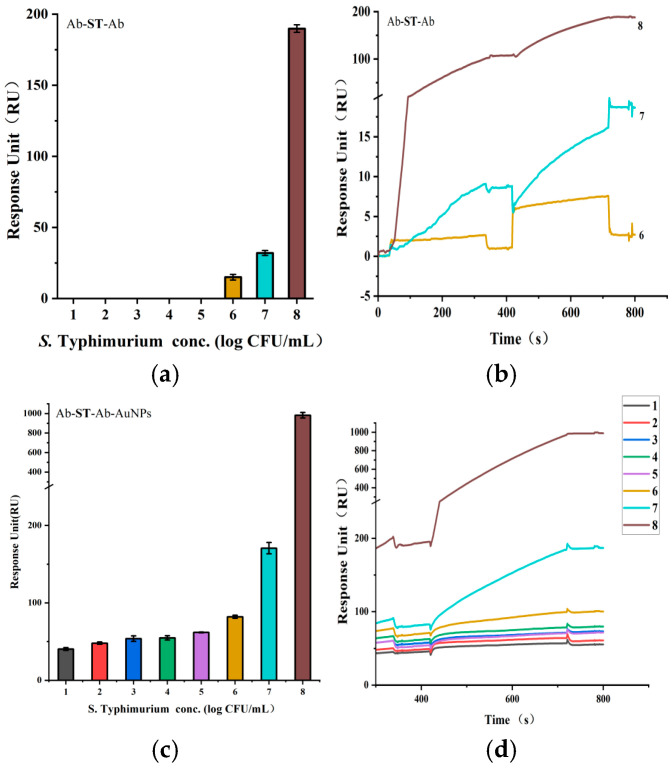
Sensitivity of SPR detection of *S*. Typhimurium using different sandwich enhancement strategies. (**a**,**b**) Ab-ST-Ab configuration; (**c**,**d**) Ab-ST-Ab-AuNP configuration. Samples 1–8 correspond to concentrations ranging from 4.2 × 10^1^ to 4.2 × 10^8^ CFU/mL.

To enhance detection sensitivity, sandwich strategies using Ab-ST-Ab and Ab-ST-Ab-AuNP configurations were applied for SPR assays (Figure 6). The Ab-ST-Ab setup produced progressively stronger signals with increasing *S*. Typhimurium concentrations (4.2 × 10^6^ to 4.2 × 10^8^ CFU/mL), lowering the LOD from 4.2 × 10^7^ to 4.2 × 10^6^ CFU/mL. Replacing the secondary antibody with AuNP-labeled antibodies (Ab-AuNPs) further amplified the SPR signal across a broader range (4.2 × 10^1^ to 4.2 × 10^8^ CFU/mL, improving the LOD by five orders of magnitude to 4.2 × 10^1^ CFU/mL). The total assay time, including the separation, was under 50 min, with SPR detection completed in approximately 12 min.

### 3.4. Specificity of Detection

**Figure 7:**
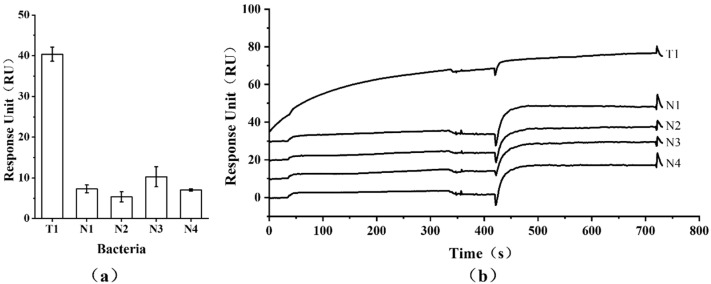
Specificity analysis of SPR detection using the Ab-ST-Ab-AuNP enhancement strategy. T1: *S*. Typhimurium (4.2 × 10^1^ CFU/mL); N1-4: *B. subtilis* natto, *S. aureus*, *B. subtilis* 168, and *E. coli* (each at 1.0 × 10^8^ CFU/mL). (**a**) Comparison of quantitative results from SPR response to different bacteria. (**b**) Alteration of SPR absorption band with different bacteria.

The specificity of the SPR assay based on the Ab-AuNP sandwich structure was clearly demonstrated. Only *S*. Typhimurium at 4.2 × 10^1^ CFU/mL exhibited a significant response of 40.38 ± 1.73 RU, while all four non-target bacteria, even at a much higher concentration of 1.0 × 10^8^ CFU/mL, yielded low or negative signals (Figure 7).

## 4. Discussion

In this study, the CE of *S.* Typhimurium captured by the IMBs exceeded 96.04% at concentrations below 4.2 × 10^3^ CFU/mL in spiked milk samples. These results are in good agreement with the previously reported average CE of 95.4% [41]. In comparison, the standard cultivation method for *Salmonella* detection typically yields an accuracy rate of 90–95% [42]. Furthermore, the low CEs of all four non-target bacteria demonstrate that the IMBs exhibited high specificity toward the target *Salmonella* cells. Although the polyclonal antibody used is specific for *S.* Typhimurium, *S.* Enteritidis, and *S.* Heidelberg, we only developed a detection system for *S.* Typhimurium, which is one of the predominant serotypes [9,38,39,40].

IMS has been combined with various detection techniques, including ELISA, LAMP, PCR [43], and SPR. IMS-SPR has been applied to detect targets like staphylococcal enterotoxin B [44], *Vibrio parahaemolyticus* [45], and other pathogens. However, achieving both low LOD and rapid results remains a challenge for SPR-based detection of pathogenic bacteria. For *Salmonella*, direct SPR detection via antigen–antibody interactions typically yields LODs between 1.4 × 10^4^ and 1.3 × 10^7^ CFU/mL [22,25,46,47]. Although a much lower LOD of 2.8 ± 19.6 CFU/mL was reported in one study, it required a prolonged 10 h on-chip pre-enrichment step [48]. In this study, the LOD for direct SPR detection was 4.2 × 10^7^ CFU/mL, highlighting its limitations for practical use in the food industry.

SPR-based sensors face sensitivity limitations in detecting pathogenic bacteria due to several factors. First, antibody-mediated capture under fluid flow must overcome hydrodynamic and shear forces [23]. Second, the evanescent field penetrates only ~300 nm from the sensor surface, while micron-sized bacteria mostly interact with the outer glucan layer. As a result, only bacteria in close proximity induce a measurable refractive index change, and larger bacteria generate weaker signals [49]. Additionally, steric hindrance prevents uniform surface coverage [50], leaving most *S.* Typhimurium cells outside the effective sensing range, meaning they contribute little to the overall signal.

SPR signals are highly sensitive to refractive index changes near the sensor surface, which are directly proportional to the mass bound. Sandwich strategies are commonly employed to enhance signal strength, as secondary binding events introduce additional mass [27]. Compared to SPR, LSPR offers greater sensitivity to local refractive index changes around nanostructures, requires simpler instrumentation without prism coupling or precise angular/wavelength control, and allows for miniaturization and cost reduction.

For example, Ko et al. used AuNP-labeled polypeptide–protein A to detect *Salmonella*, achieving a detection time of approximately 25 min and an LOD of 10^5^ CFU/mL [51]. Vaisocherová-Lísalová et al. further improved sensitivity using an AuNP–antibody sandwich structure, achieving an LOD of 11.7 × 10^3^ CFU/mL, with a detection time of 15 min and a total time of 80 min [52].

In this study, the antibody sandwich strategy reduced the LOD to 4.2 × 10^6^ CFU/mL, while incorporating AuNPs further improved sensitivity by five orders of magnitude to 4.2 × 10^1^ CFU/mL. Non-specific binding between IMBs and non-target bacteria was below 8%, and all four non-target strains at 1.0 × 10^8^ CFU/mL yielded low or negative signals, indicating minimal assay interference. This likely resulted from effective BSA and EA blocking of carboxyl groups, reducing non-specific adsorption in the SPR microfluidic systems [53]. The only comparable sensitivity was reported by Liu et al., who achieved an LOD of 1.4 × 10^1^ CFU/mL for SPR detection of *S*. *enteritidis* using immunoMNP conjugate in PBS buffer [22].

Two main factors contribute to SPR signal enhancement by AuNPs. First, AuNPs have a higher density than antibodies or *Salmonella*, leading to a greater mass change per unit volume [54]. Second, their high dielectric constant and the electromagnetic coupling between AuNPs and the metal film on the sensor surface enhance SPR sensitivity [55,56].

A major limitation of SPR detection is the high cost of sensor chips, which hinders widespread adoption. Regeneration offers a practical solution. An effective regeneration protocol must (i) remove bound *Salmonella* without compromising antibody activity and (ii) restore the SPR signal to baseline. Notably, treatment with 0.85% phosphoric acid achieved an IMB recovery efficiency of 92.32 ± 1.23%, demonstrating strong regeneration capability.

## 5. Conclusions

In conclusion, this study used immunomagnetic beads to swiftly and efficiently separate *S.* Typhimurium from PBS and milk samples. Two sandwich strategies, Ab-ST-Ab and Ab-ST-Ab-AuNPs, were adapted to amplify the SPR signal, reducing the LOD to 42 CFU/mL. The total detection time from sample collection to final SPR readout was under 50 min. These findings demonstrate the development of a rapid and sensitive assay for *S.* Typhimurium which holds significant promise for enhancing food safety, particularly in fast food contexts, and for improving traceability of foodborne salmonellosis. Future work employing this polyclonal antibody for *S.* Enteritidis and *S.* Heidelberg detection, as well as monoclonal antibodies to detect various *Salmonella* serotypes, could further support epidemiological studies and accurate source tracing.

## Data Availability

The analyzed data presented in this study are included within this article. Further data are available upon reasonable request from the corresponding author.
